# A complex network approach reveals a pivotal substructure of genes linked to schizophrenia

**DOI:** 10.1371/journal.pone.0190110

**Published:** 2018-01-05

**Authors:** Alfonso Monaco, Anna Monda, Nicola Amoroso, Alessandro Bertolino, Giuseppe Blasi, Pasquale Di Carlo, Marco Papalino, Giulio Pergola, Sabina Tangaro, Roberto Bellotti

**Affiliations:** 1 Istituto Nazionale di Fisica Nucleare (INFN), Sezione di Bari, Via A. Orabona 4, 70125 Bari, Italy; 2 Department of Basic Medical Science, Neuroscience, and Sense Organs, University of Bari, Piazza G. Cesare 11, 70124 Bari, Italy; 3 Department of Physics ‘Michelangelo Merlin’, University of Bari ‘Aldo Moro’, Via G. Amendola 173, 70126 Bari, Italy; 4 Psychiatry Unit - Bari University Hospital, Piazza G. Cesare 11. 70124 Bari, Italy; University of Texas at San Antonio, UNITED STATES

## Abstract

Research on brain disorders with a strong genetic component and complex heritability, such as schizophrenia, has led to the development of brain transcriptomics. This field seeks to gain a deeper understanding of gene expression, a key factor in exploring further research issues. Our study focused on how genes are associated amongst each other. In this perspective, we have developed a novel data-driven strategy for characterizing genetic modules, i.e., clusters of strongly interacting genes. The aim was to uncover a pivotal community of genes linked to a target gene for schizophrenia. Our approach combined network topological properties with information theory to highlight the presence of a pivotal community, for a specific gene, and to simultaneously assess the information content of partitions with the Shannon’s entropy based on betweenness. We analyzed the publicly available BrainCloud dataset containing post-mortem gene expression data and focused on the Dopamine D2 receptor, encoded by the *DRD2* gene. We used four different community detection algorithms to evaluate the consistence of our approach. A pivotal *DRD2* community emerged for all the procedures applied, with a considerable reduction in size, compared to the initial network. The stability of the results was confirmed by a Dice index ≥80% within a range of tested parameters. The detected community was also the most informative, as it represented an optimization of the Shannon entropy. Lastly, we verified the strength of connection of the *DRD2* community, which was stronger than any other randomly selected community and even more so than the Weighted Gene Co-expression Network Analysis module, commonly considered the standard approach for such studies. This finding substantiates the conclusion that the detected community represents a more connected and informative cluster of genes for the *DRD2* community, and therefore better elucidates the behavior of this module of strongly related *DRD2* genes. Because this gene plays a relevant role in Schizophrenia, this finding of a more specific *DRD2* community will improve the understanding of the genetic factors related with this disorder.

## Introduction

Converging evidence suggests that risk for complex heritable diseases is associated with several interacting genes possibly merging in molecular modules or pathways [1], whose identification is key to shed light on the biology of brain diseases. Gene co-expression implicates genetic communities that may be relevant for schizophrenia [2–4]. However, it is crucial that the number of genes in these modules, also called communities of genes, should not be too large, because modules comprising of hundreds of genes are often too populated to gain meaningful biological insights [5]. In this regard, risk for schizophrenia is associated with common polymorphisms, each adding a small effect on the probability of illness. The fact that gene expression is co-regulated and pathways are likely co-expressed strongly influences the organization of molecular pathways [6]. This may be also the case of schizophrenia genes and consequently risk genes for this illness may be linked through co-expression pathways [7–9]. In this study, we investigated brain-specific gene co-expression in a brain region crucially involved in schizophrenia, i.e., the dorsolateral prefrontal cortex to detect molecular pathways of risk genes. The *DRD2* gene coding for the D2 dopamine receptor is an optimal candidate for investigating the genetic architecture of schizophrenia-related molecular pathways because of its genome-wide association with diagnosis of this brain disorder and for its well established role in its biological underpinnings [10]. Thus, we developed a novel approach to investigate in healthy subjects a number of genes strongly linked with *DRD2*. The development and availability of an increasing number of precision techniques to quantify gene transcription challenges the field of molecular psychiatry. In this context, gene co-expression network analysis addresses the need to formalize, include and manage all the information originating from genetic data [11]. The rationale is to investigate a network whose edges are represented by correlation measurements between gene expressions, with genes being the nodes of the graph [12]. This approach integrates information related to multiple genes, rather than targeting single candidate genes. Because genes interact with each other and are co-regulated by molecular agents (e.g., transcription factors, miRNA), the investigation of gene co-expression networks yieldss greater biological plausibility than single gene studies [13]. Several approaches have been proposed to investigate gene co-expression networks. In particular, Weighted Gene Co-expression Network Analysis (WGCNA) [14] can be considered as a strategy for this study. WGCNA provides a network identification based on the similarity of genetic trascription-level profiles across individuals by defining clusters of co-expressed genes. A strength of WGCNA is that connections are graded, i.e., all genes are connected at variable degrees. This procedure enhances the sensitivity to detect weak genetic links and takes also into account the scale-free organization of known biological networks [15]. However, the clusters detected by WGCNA, called gene modules, are only partially replicable across different datasets [16]. Consequently, we proposed an unconventional application of hard threshold analysis. The standard use of WGCNA implicates loss of information and sensitivity when implementing thresholding [17]. Our main goal therefore was to detect a pivotal gene community, beginning with a WGCNA study conducted in a previous work [18] where a *DRD2* co-expression module was found. We developed additional analyses based on the study of topological properties of the detected community and its information content. For this purpose, we compared and tested our proposed method on four different community detection algorithms: Fasts Greedy, Louvain, InfoMap, Walktrap. We aimed to demonstrate that the community found using our methodology was a pivotal gene community and it emerged consistently when we applied different community detection algorithms. This community could represent a more accurate model of the co-expression interactions of the *DRD2* gene relative to the WGCNA module we previously investigated.

## 1 Materials and methods

**Fig 1:**
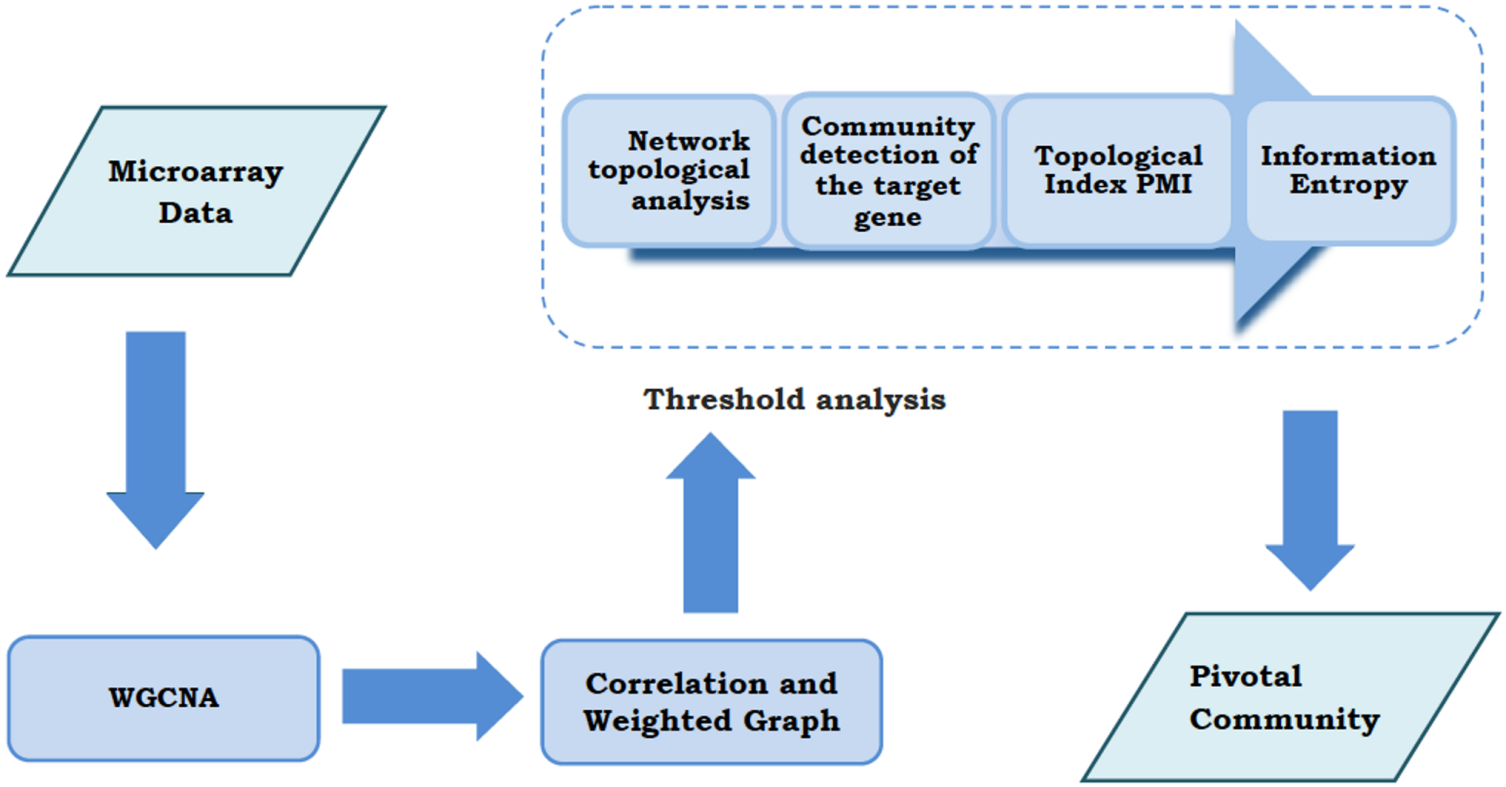
Flowchart of the methodology. After the identification of a WGCNA community with a target gene, correlation measures have been applied to build a co-expression gene network. The combined study of the network’s topological properties with information entropy, through a threshold analysis, led to the detection of a pivotal community for the target gene.

The publicly available BrainCloud dataset [19] was employed to extract gene expression data, focusing on the dopamine D2 receptor. This dataset was developed through a collaboration between the Lieber Institute for Brain Development and the National Institute for Mental Health (NIMH) and contains the post-mortem gene expression data of 268 subjects without neuropathological or neuropsychiatric diagnosis. We only selected observations with RNA integrity numbers (RIN) greater than 7.0 owing to the higher quality of the tissue sample [20]. Furthermore, Caucasian and African American subjects were included in the final sample because of the low number of observations in the Hispanic and Asian groups. The final dataset included 199 subjects with a mean age of 32 ± 20 years (range: 0–77 years). The meta-data for each subject are available in BrainCloud and include demographic variables such as age, sex and ethnicity, as well as sample quality features (RIN, pH, post-mortem interval). The novel methodological pipeline implemented consists of seven main steps, as summarized in Fig 1, which are to: (1) identify a WGCNA module including a target gene; (2) apply correlation measures to define the network of co-expressed genes; (3) study the topological properties of the network; (4) implement four community detection methods to study the structure of the community detected; (5) summarize the topological properties of the detected community in a new composite index—the Pivotal Module Index—that identifies a strategic and cohesive community for the target gene; (6) use the information theory applied to the hard threshold analysis to compute the informativeness of the community structure and confirm the findings of the topological analysis; and, lastly, (7) detect a pivotal community for the target gene and confirm its stability.

### 1.1 Identifying a WGCNA module and target gene

This step of our methodology refers to a previous work in which we used the WGCNA to investigate a dataset of 23,636 genes and characterize the co-expression network, including the long isoform of *DRD2* (i.e., D2L) [18]. The transcriptomic context of this gene was studied in order to translate the genetic variation of the *DRD2* community into imaging and clinical phenotypes. We found 84 genes that clustered with *DRD2* in a module enriched for DNA packaging, and involved in the regulation of dopamine secretion, and also in schizophrenia risk.

### 1.2 Correlation measures for a network of co-expressed genes

We implemented a hard threshold analysis on the module defined by WGCNA, containing the *DRD2* gene, to further specify the co-expression network [21]. In this way we applied WGCNA to obtain a first coarse clustering and then combined it with a deep characterization of the module of interest through the use of hard thresholding.

Starting from the 85 gene expressions *x*_*i*_, *i* = 1, …, 85, and the 199 subjects selected, we measured the absolute value of Pearson’s pairwise correlations *s*_*ij*_ to define the network of co-expressed genes [22]:

$$\begin{array}{c} s_{ij}=\left|cor\left(x_{i},x_{j}\right)\right|. \end{array}$$ (1)

We did not considered the sign of the correlation since we focused on the strength of the relationship between the pairs of genes, while we were not interested in the direction of such relationship [15]. Hence, we obtained ‘adjacency matrix *A*’, where each elements *a*_*ij*_ = *f*(*s*_*ij*_) is a function of the correlation measurements and measures the weight of the connection between two nodes of the network. This matrix formally represents the weighted graph and it is usually elaborated by applying two different thresholding methods: the soft thresholding, which is based on a power of the *s*_*ij*_ and the hard thresholding, based on the following matrix formula:

$$\begin{array}{c} a_{ij}=signum\left(s_{ij},t\right)=\{\begin{array}{cc} 1 & \text{if}\;s_{ij}\geq t\\ 0 & \text{if}\;s_{ij}<t \end{array} \end{array}$$ (2)

where *t* is the threshold value.

In this step of analysis we wished to emphasize that we had already exploited soft thresholding procedures, given that our initial network is a WGCNA module. Consequently, we investigated the possibility to further fractionating the WGCNA module by means of a hard threshold analysis.

### 1.3 Topological properties of the network

To select the best threshold value, we studied the trend of the main network’s topological properties while uniformly varying the *t* threshold. The intrinsic hypothesis is that a varying threshold for pairwise correlations can be used to highlight the existence of network communities that persist for different thresholds as well. Because the presence of a community structure affects the network properties, we expect this type of organization to clearly emerges while exploring a wide range of thresholds. Several topological properties have been investigated, namely: degree, betweenness, diameter, eccentricity and length of detected community. Briefly, the degree, *k*_*i*_, identifies the amount of connections that the node *i* has in comparison to all other nodes in the network *j*, with *j* = 1, …, *N* and it is defined as follows:

$$\begin{array}{c} k_{i}=\sum_{j\in N}a_{ij}, \end{array}$$ (3)

where *a*_*ij*_ are the elements of the adjacency matrix, *A*. The *a*_*ij*_ elements have binary or continuous values for either a soft or a hard threshold, respectively, depending on the nature of the adjacency matrix. In the first case, also known as weighted networks, this quantity is called ‘strength’. For each node *i*, it represents the sum of the weights extended to the node-connected neighbors and indicates the overall strength of the node connectivity. Betweenness, *b*_*i*_, is another centrality measure that evaluates the role of the node in connecting each other couple of genes. Thus, it emphasizes the pathway of the considered node with respect to all possible pathways in the entire network:

$$\begin{array}{c} b_{i}=\sum_{j,k\in N,j\neq k}\frac{n_{jk}\left(i\right)}{n_{jk}} \end{array}$$ (4)

In this equation *n*_*jk*_ represents the number of geodesics between node *j* and *k*, while *n*_*jk*_(*i*) is the number of geodesics between the same genes, passing through node *i*. A geodesic between two nodes *j* and *k* is defined to be the shortest path connecting a node *j* with a node *k*. Given that betweenness takes into account the level of criticality in connecting alternating pairs of nodes in the network, it can present a global view of the complex networks structure. Furthermore, we examined the diameter property *D*, which is the maximum geodesic of a graph. This can be considered the size measure of the graph itself. Another property we explored was eccentricity. For each node *i*, the eccentricity is defined as the maximum geodesic starting from node *i*. Accordingly, this can be considered a geometrical alternative measure of centrality. The last important property in community detection is the length of the detected community, i.e., the number of nodes belonging to the community. Hence, modules comprising hundreds of genes might be too general to gain biological insight, while modules with few genes typically lose the meaning of community. All these properties, with the exception of diameter, which is already a global network property, were considered on average to characterize the network behavior.

### 1.4 Module structure: Four community detection methods

To the WGCNA network identified we applied a representative set of four community detection algorithms most used in the literature; i.e., Fast Greedy, Louvain, Walktrap, and InfoMap. These algorithms have been thoroughly described in a previous work [23]. In brief, they differ on the basis of the qualitative definition of the community they adopt [24]. More specifically, these algorithms differ on the process that allows the estimation of the community structure and the measure used to quantify this clusterization as modularity, similarity or mutual information. Modularity is based on the number of intra-community and inter-community links [25–29]. It measures the quality of the partition, meaning that there are many edges within the communities and only a few between different communities [30]. Fast Greedy and Louvain are two modularity optimization algorithms, that differ in the performance of optimization. Fast Greedy [23] is based on greedy optimization, while Louvain achieves a community aggregation step that improves the performance on large networks [31]. The Walktrap algorithm exploits node similarity measures. The measure quantifies a community as an array of elements that are similar to each other, but dissimilar from the other nodes of the network. In particular Walktrap is a hierarchical agglomerative clustering method that computes similarity based on random walks [32]. The InfoMap algorithm [33] is based on Shannon’s source coding theorem [34] and it relies on the measure of mutual information. This measure quantifies how much we learn about a node, i.e. A, if we know another node B, and viceversa [35].

### 1.5 Novel network substructure metric: Pivotal Module Index

By implementing the set of four community detection algorithms mentioned above, we obtained different community structures for varying threshold values. We used a novel composite index to highlight the presence of a pivotal community. Betweenness, degree, diameter and length of the detected community were adopted as the most relevant properties to construct this topological index. The new PMI points out the presence of essential nodes. In particular, high degree and betweenness highlight the presence of intense and strategical link connections, respectively. Fairly small diameter supports the cohesiveness community. At the same time, by definition, the PMI supports the cohesive community. Consequently, it reveals the presence of a pivotal community for strategical impact and more cohesiveness within genes. The PMI was defined as following:

$$\begin{array}{c} PMI=\frac{k_{tc}\cdot b_{tc}}{D_{tc}\cdot N_{tc}} \end{array}$$ (5)

where, *k*_*tc*_, *b*_*tc*_, *D*_*tc*_, *N*_*tc*_ are the degree, betweenness, diameter and number of nodes of the target gene community, respectively. To estimate the statistical error associated with the PMI we implemented a bootstrap procedure [36]. According to this method, a set of data is randomly resampled numerous times with replacement. Thereafter, the statistical indicators, e.g. standard error or the confidence interval, are evaluated based on these new samples [37]. The data sample with 199 subjects was resampled 1000 times, and then the evaluation of the PMI was repeated. For each threshold value, a statistical distribution of the PMI was thereby obtained with 1000 estimations of PMIs. In the present analysis, we used the range between the 25*th* and 75*th* percentiles of this distribution as the statistical error estimates of the index. We then studied the community identified by means of the PMI using two independent approaches: *Information Theory* in order to evaluate if the pivotal detected community was also the most informative possible, and *Dice Index* to assess the stability of the detected co-expression community.

### 1.6 Information entropy based on betweenness

Information entropy can measure the mean information contained in a data sample, as for example in a time series or in an image [34]. Defined as, *A* = {*a*_1_, *a*_2_, *a*_3_, …, *a*_*n*_} a discrete random variable with probability mass function *P*(*A*), the information entropy is calculated using the following formula:

$$\begin{array}{c} H\left(A\right)=-\sum_{i=1}^{n}P\left(a_{i}\right)log_{2}P\left(a_{i}\right) \end{array}$$ (6)

Information entropy is an important index to describe the structure of a complex network [38] and can be used to determine the number of clusters in a data set [39, 40]. In this investigation, we used a method that evaluates the information entropy based on betweenness. There is a plethora of literature (see for example [41–43]) on the importance of betweenness for graph characterization. The novelty of this approach resides in the emphasis given to the information based on strategic power, as expressed by betweenness values. There is an extremely strong correlation between entropy per node and betweenness of the node. In particular, if we randomly choose two nodes of the network that present high betweeneess, they will have a high probability of occurring on the same shortest path. Hence, these nodes will contribute significantly to entropy production [44]. For a complex network with N nodes the entropy based on betweenness is defined as follow:

$$\begin{array}{c} H_{bet}=-\sum_{i=1}^{N}b\left(i\right)log_{2}\left[b\left(i\right)\right] \end{array}$$ (7)

where *b*(*i*) is the betweenness of *i*-*th* node defined by Eq 4. To make information content independent of module size, the information entropy of a network or a community was divided by the number of nodes of the network or community, respectively:

$$\begin{array}{c} H_{bet}=\frac{H_{bet}}{N_{tc}} \end{array}$$ (8)

A system with maximum entropy is a system with maximum information content [45]. Accordingly, an optimal community will be characterized by a high informational entropy value.

### 1.7 Pivotal community for the target gene and its stability

#### 1.7.1 Dice index

A pivotal *DRD2* community was later detected during our analysis, and in step 7 of the pipeline we confirmed the stability of our results using the Dice index. This index is a statistic measure used for comparing the similarity of two samples [46]. Considering two sets *A* and *B*, Dice index quantifies the overlap between them. It is defined as:

$$\begin{array}{c} Dice=\frac{2\;\cdot |(A\cap B)|}{|A|+|B|} \end{array}$$ (9)

where ∩ is the intersection. The Dice index changes in a restricted range of values [0, 1]. In this work, we used Dice index to measure the overlap between different communities. A Dice index equal to 1, computed on two communities, means that they are exactly the same. Conversely, a Dice Index equal to 0 indicates that they have no elements in common. We used the index for two purposes:

1. to compare results obtained through four community detection methods described in section 1.4;
2. to verify the stability of detected gene community.

For the point 1. Dice was computed using the *DRD2* community, founded by the Fast Greedy method, as the reference one, and the other three implemented community detection techniques. For the second point, if we analyze a community over different condition, e.g. varying threshold values, and verify high index values, we can assert that the selected community is stable against the choice of threshold. Once the best threshold *T* is selected, we computed:

$$\begin{array}{c} Dice_{i}=\frac{2\;\cdot |(C_{T}\cap C_{i})|}{|C_{T}|+|C_{i}|} \end{array}$$ (10)

where *C*_*T*_ is the gene community of *DRD2* found for the best threshold chosen, and *C*_*i*_ is the gene community of *DRD2* which emerged for different threshold values belonging to the neighborhood *T*.

#### 1.7.2 Strength of the detected community

After validating the information content and the stability of the detected community, we measured the embeddedness of the *DRD2* gene in its community. In other words, we set out to verify if *DRD2* is more connected in selected communities than in an entire network or in a random module. Strength, as a graph property, has been defined in section 1.3. The strength of *DRD2* is a natural property for investigating the relationship between neighboring *DRD2* genes. We compared the strength of *DRD2* for the resulting WGCNA module with the strength of *DRD2* for the detected community. We also calculated the strength for 1000 random repetitions of the *DRD2* community. The strength values were divided by the number of community nodes.

## 2 Results and discussion

### 2.1 Identifying a WGCNA module and target gene

**Fig 2:**
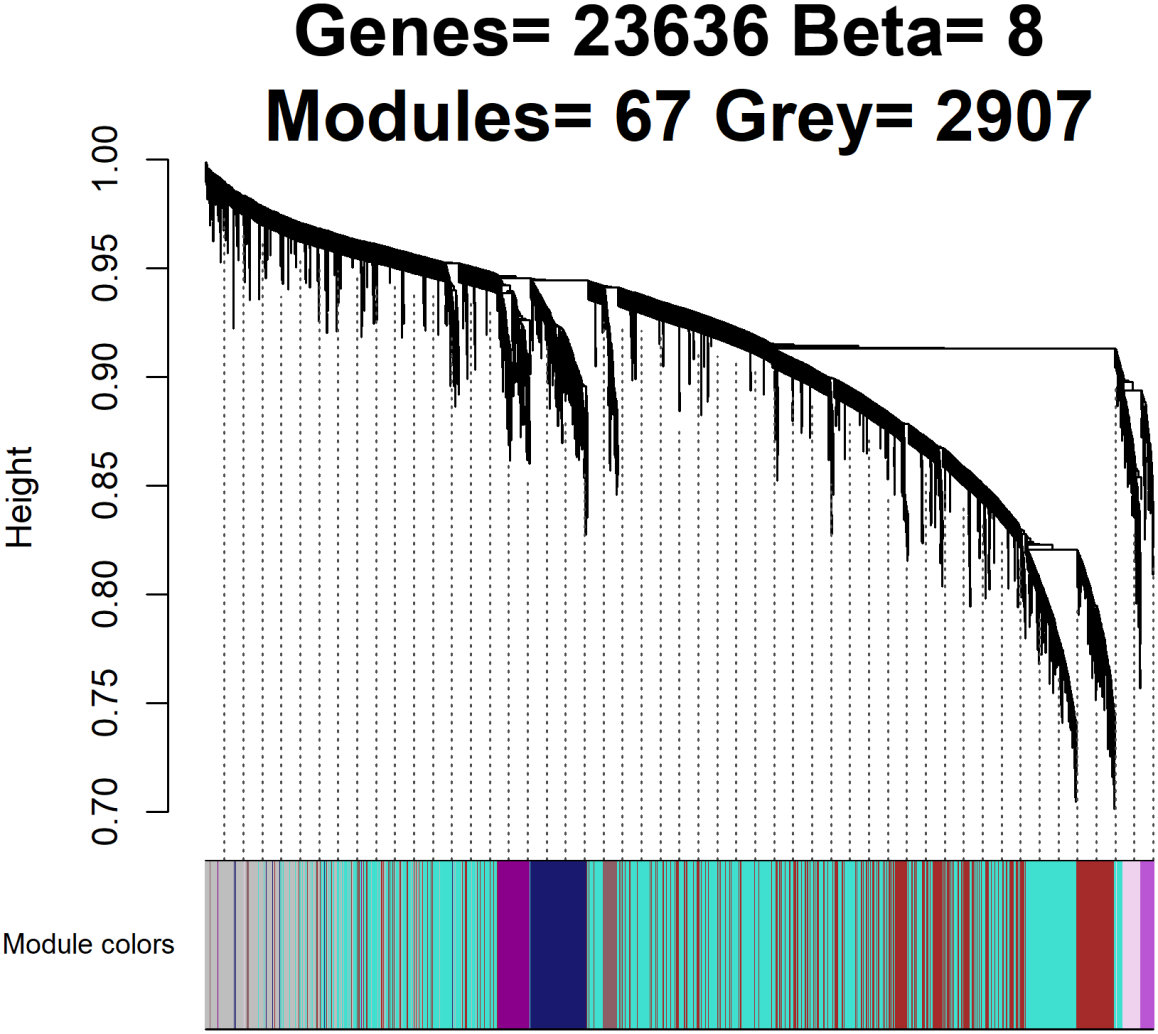
Dendrogram of the network with defined module colors. This dendrogram was obtained by average linkage hierarchical clustering. The color spectrum underneath the dendrogram indicates the module assignment determined by the Dynamic Tree Cut. The *DRD2* gene is contained in the maroon module. Almost 3000 genes are isolated (grey module).

Fig 2 shows a dendrogram of 67 modules found by means of the WGCNA algorithm. The *DRD2* gene is contained in the maroon module computed in a previous work on the BrainCloud dataset [18].

### 2.2 Correlation measures for a network of co-expressed genes

**Fig 3:**
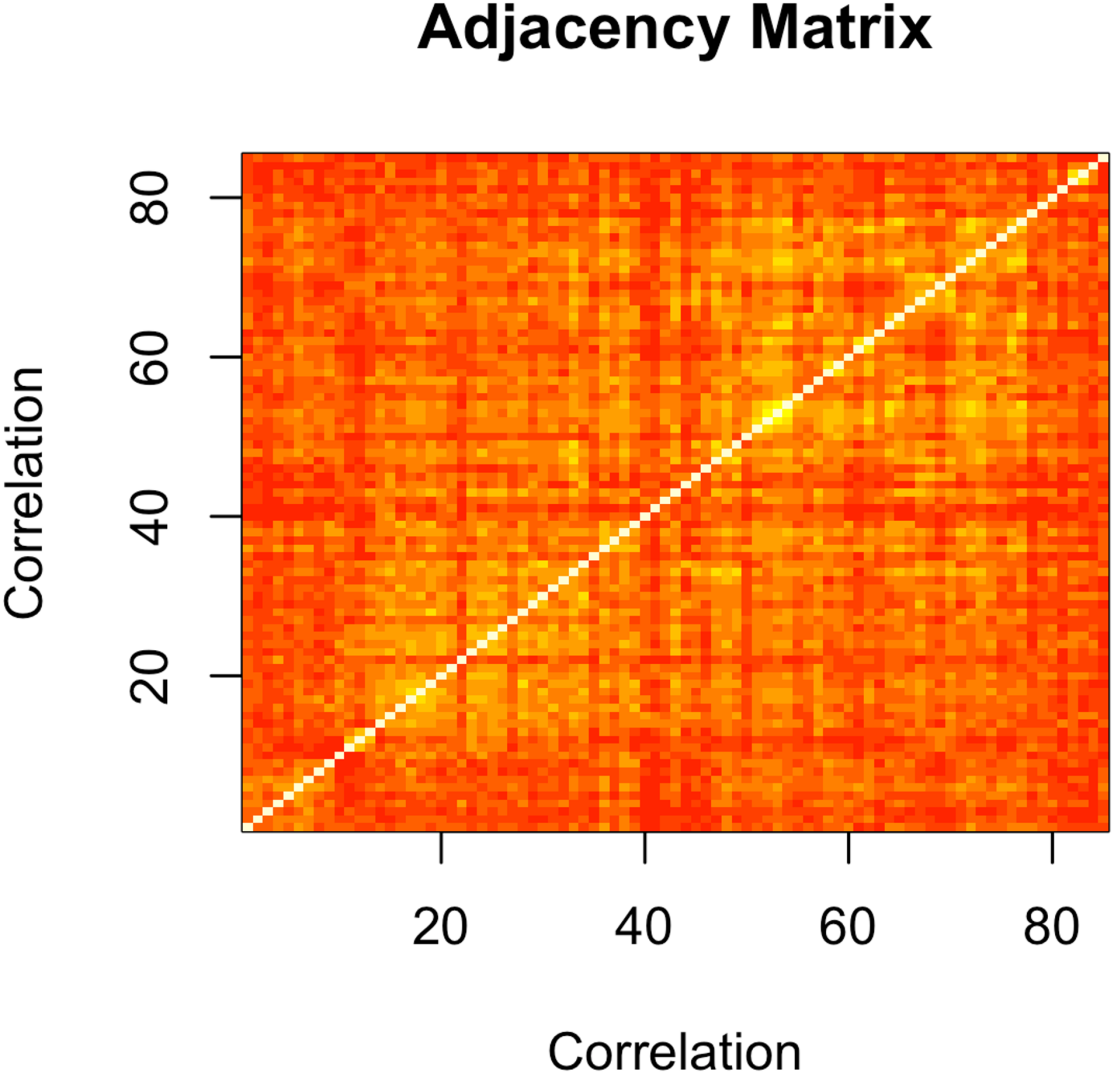
Adjacency matrix of the maroon module. This matrix illustrates the similarity between gene expressions using the correlation matrix. Color gradient indicates the strength of the connections between genes.

Fig 3 illustrates the adjacency matrix of the maroon module after applying the correlation metric.

### 2.3 Topological properties of the network

**Fig 4:**
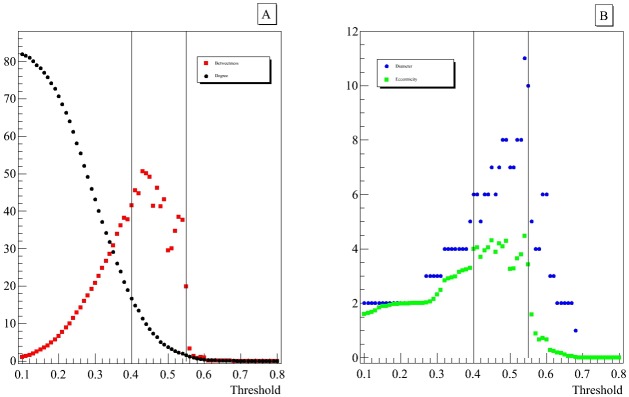
Trend of the main topological properties of the network. The vertical lines highlight an optimal range of threshold values between 0.40 and 0.55 in which three of the four network properties are maximized. For visual clarity, threshold values in which the topological properties are not significant have been omitted.

Fig 4 represents four varying network properties (see 1.3) at different threshold values. The network reveals an interesting tendency in a limitated threshold ranges (between 0.4 and 0.55) in which three topological properties of the network are maximized. For lower threshold values we observe only monotonic trends due to the reduction in the number of links and it is reasonable to assume noisy relationships until the detected range. On the contrary for higher correlations the loss of information is too much, in fact, all the properties tend towards zero. This range was chosen to search for a smaller and stable community including the *DRD2* gene.

### 2.4 PMI and community detection methods

**Fig 5:**
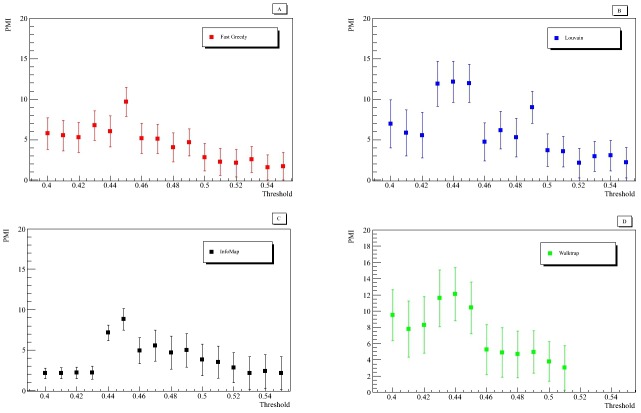
Pivotal Module Index, as a function of threshold. Pivotal Module Index as a function of threshold in the optimal range of the network topological properties obtained using four different community detection methods. Statistical errors were estimated by means of the bootstrap procedure.

By definition, high PMI values correspond to the presence of pivotal communities. We plotted the PMI, Fig 5, for several threshold values to obtain a distribution that indicates the thresholds at which the most strategic and cohesive communities could be found. The PMI was computed on the *DRD2* communities. Fig 5 displays the PMI used for the different community detection methods as a function of threshold in the previously selected range. We only plotted modules that contained at least three genes, thus preserving the concept of community, which was adopted in other community detection studies as the international DREAM challenge (https://www.synapse.org/#!Synapse:syn6156761/wiki/).

**Fig 6:**
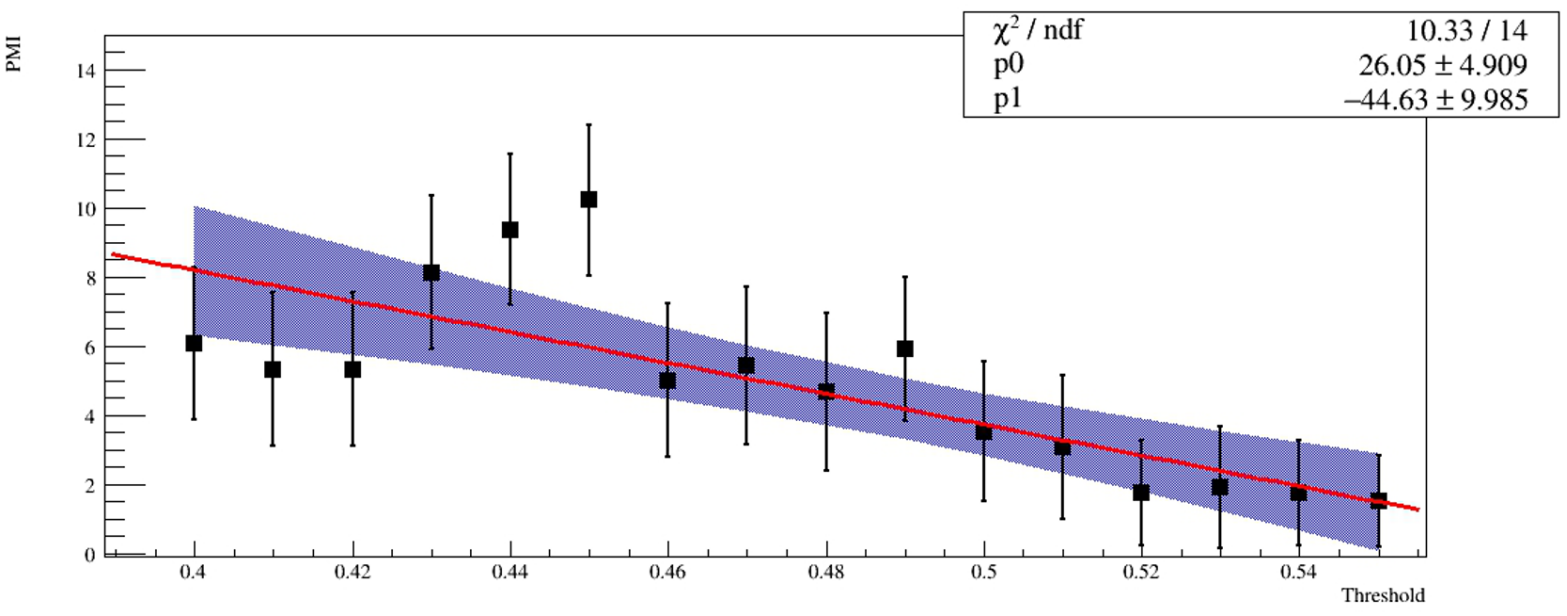
Average PMI distribution. Average Pivotal Module Index (PMI) distribution for the four community detection methods fitted with a linear model. The blue area represents 95% confidence intervals for the fitted function. Statistical errors were estimated by means of the bootstrap procedure. In the top right corner the reduced *χ*^2^ and fit parameters are been shown.

The distributions displayed in Fig 5 present a local or absolute maximum value equal to 0.45 that appears to be a good choice for the threshold. According to the definition of the PMI, this implies that the structures of *DRD2* communities detected using the four different methods are approximately the same. Table 1 highlights the results obtained for *DRD2* community with a threshold value equal to 0.45 by means of the selected detection methods. The performance of each algorithm was expressed in terms of Dice index. According to PMI distributions and the Dice index values, always greater or equal to 0.88, we inferred that the methods applied are consistent amongst each other. This led us to surmise that the *DRD2* community detected by these algorithms was a pivotal community of the network and it existed and emerged irrespectively of the algorithms implemented. Moreover, Fig 6 displays the average PMI distribution of the four community detection methods fitted with a linear model. For the linear function estimated, the *χ*^2^ goodness-of-fit test implemented presents a p-value that rejects the null hypothesis at 5% level. The blue area represents 95% confidence intervals for the fitted function. The distribution index for a threshold value equal to 0.45 appears significantly higher than the linear fit. This means that a threshold value equal to 0.45 clearly outperforms all the possible thresholds in determining a preferred community. List of genes obtained through the four different community detection algorithms at threshold value 0.45 is given in the S1 Table. A strong overlap between all these methods emerges, as we reported in Table 1 with the Dice analysis. Therefore, for the sake of simplicity, hereafter we will use the Fast Greedy method to define the *DRD2* community. The community includes gene-gene pairs for which independent evidence of co-expression and gene interactions in literature has been reported, see S2 Table. Moreover, we investigated the presence of hub genes in the new detected community with respect to the original network. For the whole network, we considered as hub gene all the genes that have a scaled strength greater than 0.70. The scaled strength is the strength of the genes divided by the maximum value of strength in the network. Table 2 reports the hub genes of the whole network and the overlap with the list of genes of the detected community: 70% of the hub genes in the original module belong to the new *DRD2* community.

**Table 1:** Results of the *DRD2* community obtained using four community detection algorithms. The number of genes for the *DRD2* community found applying four different community detection algorithms at the selected threshold. The index values next to 1 suggest that the four algorithms are consistent amongst each other and the communities found are similar. Statistical errors were estimated by means of the bootstrap procedure.

Methods	number of genes	Dice
Fast Greedy	28 ± 4	1
Louvain	22 ± 4	$0.880_{-0.020}^{+0.010}$
InfoMap	30 ± 6	$0.966_{-0.026}^{+0.034}$
Walktrap	27 ± 6	$0.981_{-0.04}^{+0.01}$

**Table 2:** Hub genes list of the whole network. The hub genes of the original module [18]. The first column reports the probe name in BrainCloud. The second column reports the corresponding gene name. The third column contains the strength of hub genes. The fourth column reports the strength of hub genes divided by the maximum value of strength. Colored rows indicate the overlap with the list of genes of detected *DRD2* community.

OligoID	Gene	Strength	scaled strength
hHA034464	IGSF1	3.240	1
hHA034560	TTN	2.907	0.897
hHC022740	CLDN4	2.853	0.881
hHA039264	GATAD2A	2.819	0.870
hHA033312	CHIA	2.590	0.799
hHC025044	SDK2	2.494	0.770
hHR025236	OR2S2	2.467	0.761
hHA039456	NEURL4	2.436	0.752
hHR028896	DEFB108B	2.415	0.745
hHA034272	MAP4	2.290	0.707

The detected pivotal community for the threshold value equal to 0.45 will be analyzed with an information theory procedure in the following section.

### 2.5 Information entropy based on betweenness

**Fig 7:**
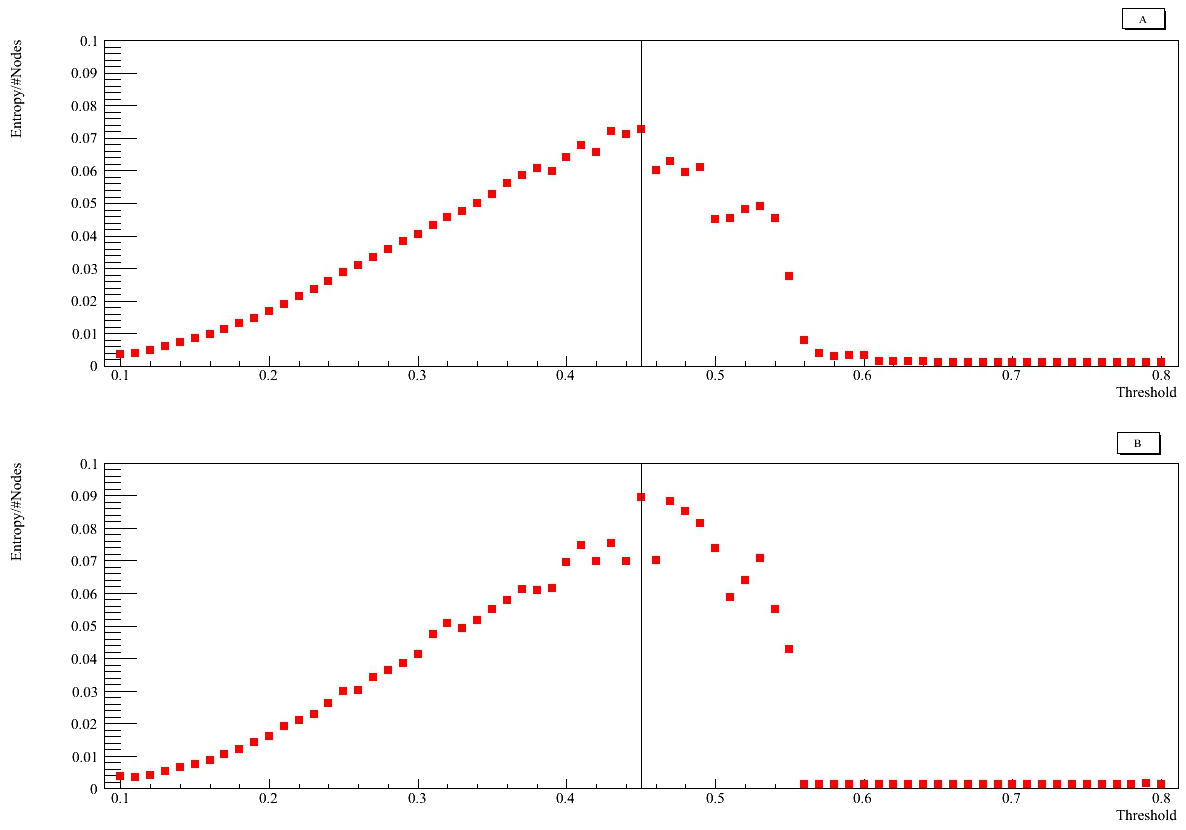
Betweenness entropy as a function of threshold. Information entropy based on betweenness as a function of threshold for the network (panel A) and for the *DRD2* community (panel B). Both distributions present a maximum threshold value equal to 0.45.

Entropy distribution, as a function of the threshold for the entire network (panel A) and for the *DRD2* community (panel B), is presented in Fig 7. *DRD2* communities were computed using Fast Greedy algorithm. The distributions for both the network and the *DRD2* community present a maximum threshold value equal to 0.45. Therefore, the more cohesive and strategic community of *DRD2*, identified in the previous section, is also the community with the highest informative significance. Hence, the selection of the threshold value equal to 0.45 is confirmed.

### 2.6 Pivotal community for the target gene and its stability

The *DRD2* community found in section 2.4 composed by 28 genes represents a consistent reduction of gene content in the WGCNA module (≥70%) and in the original BrainCloud dataset (>99%). Since we evaluated that the informativeness was maximized for this module, as for the network, we deduced that this wide reduction enhanced the biological insight within *DRD2* community. In [18] the functional enrichment of the original WGCNA module of 85 genes has been computed. Here, for the new detected community we are aware that a number of 28 genes probably represents a small set in which we are unlikely to identify more than a handful hits for each ontology. In fact, none of the functional labels identified survives Bonferroni correction. Nevertheless, the gene ontology “negative regulation of dopamine secretion” approaches significance with corrected p-value = 0.06.

Next, we verified the stability of the *DRD2* community of 28 genes.

### 2.7 Stability of the chosen community using the Dice index

**Fig 8:**
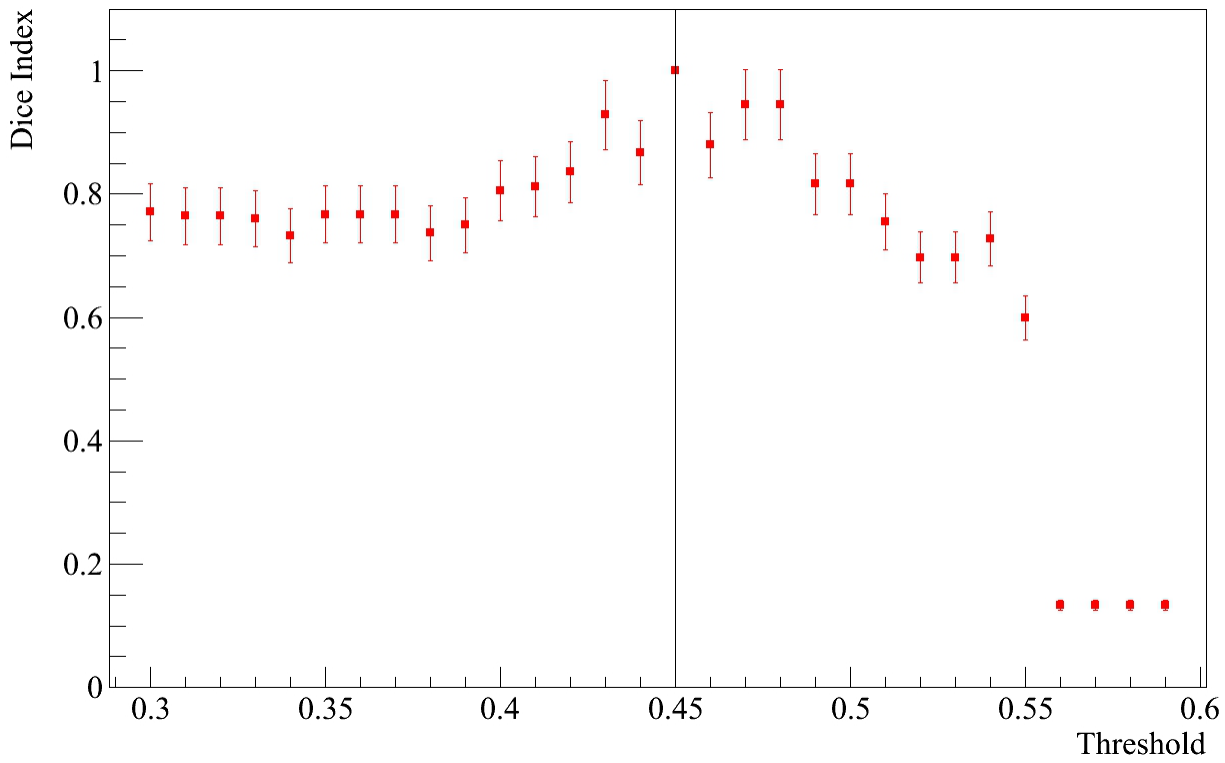
Dice index for the *DRD2* community. The Dice index as a function of threshold calculated in relation to the *DRD2* community obtained with a threshold value equal to 0.45. The statistical errors were estimated by means of the bootstrap procedure.

We computed the Dice index (Eq 10), where *T* = 0.45 and *i* is a threshold value between 0.3 and 0.6 to evaluate the stability of the *DRD2* community. Fig 8 displays the Dice index computed in relation to the *DRD2* community obtained with a threshold value equal to 0.45. Moreover, the statistical errors were estimated using the bootstrap procedure with 1000 resamplings.

The stability of the community Dice distribution in the evaluated range highlights the existence of a robust core component of genes within the target community.

The *DRD2* community was seemingly composed of about the same genes and its composition remained stable at different threshold values. The detected community remained unchanged and performed a stability of more than 80% in the neighborhood, despite the different community lengths for different threshold points.

### 2.8 Strength of the detected community

In this section we applied the procedure described in section 1.7.2. Every random module was composed by *DRD2* with 27 randomly chosen genes. Table 3 reports the results of the strength of *DRD2* per node for the whole network for the *DRD2* community selected and for the 1000 randomly selected *DRD2* communities. *DRD2* was significantly more connected in the detected community by comparison with the random communities or the resulting WGCNA module.

**Table 3:** Results of the strength of *DRD2*. Strength of *DRD2* per node for the entire network, the *DRD2* community selected and for the 1000 randomly selected *DRD2* communities.

Module	Strength
Whole network	0.34
Detected *DRD2* community	0.41
Random sample	0.33 ± 0.01

### 2.9 Discussion

In the present work, we aimed to delineate a novel community detection method that improved the performance of WGCNA to demonstrate that the community found with the proposed approach was stable with a moderate number of genes. In fact, we supported the notion that the *DRD2*-detected community using four different community detection algorithms. Our approach detected a stable *DRD2* community that represented a consistent reduction of genes when compared with the WGCNA module (≥70%) and the original BrainCloud dataset (>99%). To assess the robustness of the methodology from two distinct perspectives, we adopted: (i) a new topological index—the PMI—that highlights the presence of a pivotal community; and (ii) the information entropy based on betweenness to ensure the detected community was informative. The identified *DRD2* community exhibited the same structure for all the set of community detection algorithms applied. Once we made certain the community was meaningful, we assessed the stability of the results with Dice index to confirm that the pivotal community remained stable for all the neighborhood points. We verified that the detected community still remained unchanged and gained a stability performance of more than 80% in a neighborhood of tested thresholds. Finally, we aimed to compare the detected community with other possible partitions. Consequently, we determined that *DRD2* was more strongly related to his neighborhood in the pivotal community compared with the WGCNA module and lastly with a distribution of randomly selected communities by evaluating the relative community strength. Furthermore, the *DRD2* gene appeared to be more connected in the detected pivotal community with respect to the other communities analyzed. According to the Information Theory this stable community was also the most informative. The new detected community includes 70% of the hub genes participating in the original module. We interpreted this finding as evidence that we filtered out poorly informative genes. One limitation of the WGCNA approach is that it is not always accurate in detailing the module of genes effectively; small gene communities may in some cases be included into larger modules, leading to a loss of granularity in the information content of the modules [47]. Since our method mainly focused on informative genes, it overcomes the previous mentioned limitation. The module size reduction is crucial in biological systems because it supports the gain of physiological insight. Enrichment analysis in the detected pivotal community does not reveal further insight into the biological significance of this community, likely because the number of genes, and thus the number of hits we could obtain, is very limited. The detected community could help to understand the mechanisms of several psychiatric disorders, such as Schizophrenia. For example, it is notable that the community we investigated here further supports the strong relationship between *DRD2* and the *CNR1* gene coding for the cannabinoid receptor *CB1*. Previous evidence of interaction between these two receptors and the genes coding for them has been related with cannabis use and with intermediate phenotypes of schizophrenia [48, 49]. This link is relevant also because cannabis use is a relevant environmental risk factor for schizophrenia [50], and *DRD2* genetic variation represents a genetic risk factor for the same disorder [51]. Therefore, our study extends the current literature by suggesting that these two genes are part of a co-expressed, and thus possibly co-regulated, biological pathway relevant to substance abuse and to schizophrenia. Clearly, a clinical validation is fundamental to understand whether the detected *DRD2* community carries a biological meaning. In this sense, a possibility could be the study of intermediate phenotypes.

## 3 Conclusions

In this paper we designed a data-driven method based on complex network analyses and successfully detected a pivotal community for *DRD2*, a relevant gene linked to Schizophrenia. Beginning with a common network analysis output, based on the well-known Weighted Gene Co-expression Network Analysis, we adopted the proposed strategy and found a cluster of genes that appears cohesive, informative and stable, tested by several different approaches: topological measures, Shannon entropy based on betweeneess, and bootstrap procedure. These results suggested the possibility of exploiting the topological properties and the information theory of a graph to reduce the complexity of the gene co-expression networks and focus on gene communities. These communities should be more strategic, high connected and informative, with a reduced number of expressed genes to handle and a stronger noise-free signal. Our research could be further developed according to this line of reasoning, most notably by continuing the biological validation of gene communities and investigating their relevance at the level of neuronal function, brain networks and behavior linked with brain disorders [52].

## Supporting information

S1 Table List of genes obtained through four different community detection algorithms. (PDF)

S2 Table Indipendent evidence for gene-gene pairs of co-expression and genetic interactions in literature. (PDF)
