# Supplementary material for: A complex network approach reveals a pivotal substructure of genes linked to schizophrenia
Source: PLoS One. 2018 Jan 5;13(1):e0190110. doi: 10.1371/journal.pone.0190110 (PMC5755767; doi:10.1371/journal.pone.0190110)
Supplement: S1 Table — (PDF) [file pone.0190110.s001.pdf]

S1 Table. List of genes obtained through four different community detection algorithms . The first column reports the probe name in Braincloud. The second column reports the corresponding gene name.

OligoID	Gene	Fast Greedy	Louvain	InfoMap	Walktrap
hHC020532	CPLP	X	X	X	X
hHC025044	SDK2	X	X	X	X
hHC025152	TIGD1	X	X	X	X
hHR025236	OR2S2	X		X	X
hHC028608	PCBD2	X		X	X
hHR031476	TAS2R42	X		X	X
hHA033312	CHIA	X		X	X
hHA034164	RNF128	X	X	X	X
hHA034272	MAP4	X	X	X	X
hHA034368	ARSB	X	X	X	X
hHA034464	IGSF1	X	X	X	X
hHA034560	TTN	X	X	X	X
hHA034656	EFCAB6	X		X	X
hHA034644	TTN	X	X	X	X
hHA034944	RBM6			X	
hHA035508	USH2A	X	X	X	X
hHA035616	DRD2	X	X	X	X
hHA035604	PTPN7	X	X	X	X
hHA035796	DHX9	X	X	X	X
hHA035904	DNAH9	X	X	X	X
hHA035892	GPLD1	X	X	X	
hHA035988	CNR1	X	X	X	X
hHA036180	ING1	X	X	X	X
hHA038868	HS6ST2	X	X	X	X
hHA039264	GATAD2A	X	X	X	X
hHA039456	NEURL4	X		X	X
hHA039552	TTN			X	
hHA040020	LTBP1	X	X	X	X
hHA040404	JPH2	X	X	X	X
hHA040704	DAZAP1	X	X	X	X
