# Supplementary material for: A complex network approach reveals a pivotal substructure of genes linked to schizophrenia
Source: PLoS One. 2018 Jan 5;13(1):e0190110. doi: 10.1371/journal.pone.0190110 (PMC5755767; doi:10.1371/journal.pone.0190110)
Supplement: S2 Table — (PDF) [file pone.0190110.s002.pdf]

S2 Table. Indipendent evidence for gene-gene pairs of co-expression and genetic interactions in literature. The table shows indipendent evidence for gene-gene pairs of co-expression and genetic interactions in literature. All the genes refer to the *DRD2* detected community.

Gene 1	Gene 2	Type	Source
USH2A	EFCAB6	Co-expression	Smirnov-Cheung-2009 [1]
OR2S2	USH2A	Co-expression	Smirnov-Cheung-2009 [1]
PTPN7	TTN	Co-expression	Smirnov-Cheung-2009 [1]
JPH2	EFCAB6	Co-expression	Smirnov-Cheung-2009 [1]
GATAD2A	MAP4	Co-expression	Smirnov-Cheung-2009 [1]
DAZAP1	GATAD2A	Co-expression	Smirnov-Cheung-2009 [1]
TTN	MAP4	Co-expression	Perou-Botstein-1999 [2]
GATAD2A	DHX9	Co-expression	Perou-Botstein-1999 [2]
RNF128	IGSF1	Co-expression	Mallon-McKay-2013 [3]
USH2A	TAS2R42	Co-expression	Mallon-McKay-2013 [3]
OR2S2	ARSB	Co-expression	Dobbin-Giordano-2005 [4]
PTPN7	ARSB	Co-expression	Dobbin-Giordano-2005 [4]
JPH2	EFCAB6	Co-expression	Dobbin-Giordano-2005 [4]
ING1	SDK2	Co-expression	Ramaswamy-Golub-2001 [5]
ING1	IGSF1	Co-expression	Ramaswamy-Golub-2001 [5]
PTPN7	IGSF1	Co-expression	Burington-Shaughnessy-2008 [6]
PTPN7	IGSF1	Co-expression	Wu-Garvey-2007 [7]
GATAD2A	NEURL4	Co-expression	Innocenti-Brown-2011 [8]
EFCAB6	PCBD2	Genetic Interactions	Lin-Smith-2010 [9]
CNR1	EFCAB6	Genetic Interactions	Lin-Smith-2010 [9]
CNR1	DRD2	Genetic Interactions	Lin-Smith-2010 [9]
IGSF1	DRD2	Genetic Interactions	Lin-Smith-2010 [9]
IGSF1	DNAH9	Genetic Interactions	Lin-Smith-2010 [9]
RNF128	MAP4	Genetic Interactions	Lin-Smith-2010 [9]
ING1	DRD2	Genetic Interactions	Lin-Smith-2010 [9]
ING1	IGSF1	Genetic Interactions	Lin-Smith-2010 [9]
TTN	GPLD1	Genetic Interactions	Lin-Smith-2010 [9]
USH2A	TTN	Genetic Interactions	Lin-Smith-2010 [9]
LTBP1	DRD2	Genetic	Lin-Smith-2010 [9]

		Interactions	
LTBP1	DNAH9	Genetic Interactions	Lin-Smith-2010 [9]
LTBP1	IGSF1	Genetic Interactions	Lin-Smith-2010 [9]
ARSB	GPLD1	Genetic Interactions	Lin-Smith-2010 [9]
DHX9	PCBD2	Genetic Interactions	Lin-Smith-2010 [9]
PTPN7	DRD2	Genetic Interactions	Lin-Smith-2010 [9]

References

1. Smirnov, D. A., Morley M., Shin E., Spielman R. S., Cheung V. G. Genetic analysis of radiation-induced changes in human gene expression. Nature. 2009 May 28;459(7246): 587-91.
2. Perou, C. M., Jeffrey, S. S., van de Rijn, M., Rees, C. A., Eisen, M. B., Ross, D. T., Pergamenschikov, A., Williams, C. F., Zhu, S. X., Lee, J. C. , Lashkari, D., Shalon, D., Brown, P. O., Botstein, D. Distinctive gene expression patterns in human mammary epithelial cells and breast cancers. Proc Natl Acad Sci U S A. 1999 Aug 3; 96(16):9212-7.
3. Mallon, B.S., Chenoweth, J. G., Johnson, K. R., Hamilton, R. S., Tesar, P. J., Yavatkar, A. S., Tyson, L. J., Park, K., Chen, K. G., Fann, Y. C., McKay, R. D. StemCellDB: the human pluripotent stem cell database at the National Institutes of Health. Stem Cell Res. 2013 Jan;10(1): 57-66.
4. Dobbin, K. K., et al. Interlaboratory comparability study of cancer gene expression analiysis using oligonucleotide microarrays. Clin Cancer Res. 2005; 11: 565-572.
5. Ramaswamy, S., Tamayo, P., Rifkin, R., Mukherjee, S., Yeang, C. H., Angelo, M., Ladd, C., Reich, M., Latulippe, E., Mesirov, J. P., Poggio, T., Gerald, W., Loda, M., Lander, E. S., Golub, T. R. Multiclass cancer diagnosis using tumor gene expression signatures. Proc Natl Acad Sci U S A. 2001; 98(26):15149-54.
6. Burington, B., Barlogie, B., Zhan, F., Crowley, J., Shaughnessy, J. D. Tumor cell gene expression changes following short-term in vivo exposure to single agent chemotherapeutics are related to survival in multiple myeloma. Clin Cancer Res. 2008; 14: 4821-4829.
7. Wu, X., Wang, J., Cui, X., Maianu, L., Rhees, B., Rosinski, J., So, W. V., Willi, S. M., Osier M. V., Hill, H. S., Page, G. P., Allison, D. B., Martin, M., Garvey, W. T. The effect of insulin on expression of genes and biochemical pathways in human skeletal muscle. Endocrine. 2007; Feb;31(1): 5-17.
8. Innocenti, F., Cooper, G. M., Stanaway, I. B., Gamazon, E. R., Smith, J. D., Mirkov, S., Ramirez, J., Liu, W., Lin, Y. S., Moloney, C., Aldred, S. F., Trinklein, N. D. , Schuetz, E., Nickerson, D. A., Thummel, K. E., Rieder, M. J., Rettie, A. E., Ratain, M. J., Cox, N. J., Brown, C. D. Identification, replication, and functional fine-mapping of expression quantitative trait loci in primary human liver tissue. PLoS Genet. 2011 May;7(5):e1002078.
9. Lin, A., Wang R. T., Ahn, S., Park, C. C., Smith D. J. A genome-wide map of human genetic interactions inferred from radiation hybrid genotypes. Genome Res. 2010 Aug; 20(8): 1122–1132.
